# High barley intake in non-obese individuals is associated with high natto consumption and abundance of butyrate-producing bacteria in the gut: a cross-sectional study

**DOI:** 10.3389/fnut.2024.1434150

**Published:** 2024-10-31

**Authors:** Satoko Maruyama, Tsubasa Matsuoka, Koji Hosomi, Jonguk Park, Haruka Murakami, Motohiko Miyachi, Hitoshi Kawashima, Kenji Mizuguchi, Toshiki Kobayashi, Tadao Ooka, Zentaro Yamagata, Jun Kunisawa

**Affiliations:** ^1^Department of Research and Development, Hakubaku Co., Ltd., Yamanashi, Japan; ^2^Laboratory of Vaccine Materials and Laboratory of Gut Environmental System, Microbial Research Center for Health and Medicine, National Institutes of Biomedical Innovation, Health and Nutrition, Osaka, Japan; ^3^Artificial Intelligence Center for Health and Biomedical Research, National Institutes of Biomedical Innovation, Health and Nutrition, Osaka, Japan; ^4^Department of Physical Activity Research, National Institutes of Biomedical Innovation, Health and Nutrition, Osaka, Japan; ^5^Institute for Protein Research, Osaka University, Osaka, Japan; ^6^Department of Health Sciences, School of Medicine, University of Yamanashi, Yamanashi, Japan; ^7^Department of Microbiology and Immunology, Kobe University Graduate School of Medicine, Kobe, Japan; ^8^Graduate Schools of Medicine, Osaka University, Osaka, Japan; ^9^Graduate School of Pharmaceutical Sciences, Osaka University, Osaka, Japan; ^10^Graduate Schools of Science, Osaka University, Osaka, Japan; ^11^Graduate School of Dentistry, Osaka University, Osaka, Japan; ^12^International Vaccine Design Center, The University of Tokyo, Tokyo, Japan; ^13^Research Organization for Nano and Life Innovation, Waseda University, Tokyo, Japan

**Keywords:** barley, dietary fiber, gut microbiota, obesity, natto

## Abstract

**Objective:**

Barley, abundant in *β*-glucan, a soluble dietary fiber, holds promise in obesity prevention. Given the microbial metabolism of dietary fiber in the gastrointestinal tract, we investigated the role of gut microbiota in non-obese individuals consuming high levels of barley.

**Methods:**

Our study enrolled 185 participants from “The cohort study on barley and the intestinal environment (UMIN000033479).” Comprehensive physical examinations, including blood tests, were conducted, along with separate assessments of gut microbiome profiling and dietary intake. Participants were categorized into high and low barley consumption groups based on the median intake, with non-obese individuals in the high intake group identified as barley responders while participants with obesity were designated as non-responders. We compared the relative abundance of intestinal bacteria between these groups and used multivariate analysis to assess the association between intestinal bacteria and barley responders while controlling for confounding factors.

**Results and discussion:**

Among the fermented food choices, responders exhibited notably higher consumption of natto (fermented soybeans) than non-responders. Moreover, after adjusting for confounders, *Butyricicoccus* and *Subdoligranulum* were found to be significantly more prevalent in the intestines of responders. Given natto’s inclusion of *Bacillus subtilis*, a glycolytic bacterium, and the butyrate-producing capabilities of *Butyricicoccus* and *Subdoligranulum*, it is hypothesized that fiber degradation and butyrate production are likely to be enhanced within the digestive tract of barley responders.

## 1 Introduction

The global population with obesity is continuously increasing. According to the World Health Organization, in 2022, 43% of adults worldwide were either overweight or obese, with a body mass index (BMI) greater than 25 kg/m^2^ (1). The Japanese obesity guidelines define obesity as a BMI of 25 kg/m^2^ or greater. As of 2019, 33.0% of adult men and 22.3% of adult women were reported to be obese in Japan (2, 3). The underlying cause of obesity is an energy imbalance between calories consumed and calories expended. Recent increases in high-fat and high-sugar food intake and decreases in physical activity have contributed to an increase in the obese population (1). Obesity is a significant risk factor for various non-communicable diseases, including type 2 diabetes (T2DM), hyperlipidemia, cardiovascular disease, and several cancers (such as endometrial, breast, ovarian, prostate, liver, gallbladder, kidney, and colon) therefore, it needs to be corrected (4–6). Obesity can also be prevented or corrected by making dietary changes, such as limiting energy intake and increasing the consumption of fruits, vegetables, legumes, whole grains, and nuts, in addition to regular physical activity (150 min/week for adults) (1). Barley, a grain rich in soluble fiber, *β*-glucan, has garnered attention for its reported benefits in ameliorating abnormalities in glucose and lipid metabolism, as evidenced by numerous studies (7–9). Based on the evidence, barley may contribute in treating obesity. A randomized controlled trial comprising 100 Japanese participants with a BMI of 24 kg/m^2^ or greater revealed that consumption of barley high in *β*-glucan led to reductions in both body weight and BMI (10). Thus, barley has emerged as a functional food with the potential to counteract obesity by maintaining the homeostasis of energy metabolism.

In contrast, the highly individualized nature of the dietary effects on the host was demonstrated in the PREDICT 1 study, which observed individual postprandial responses to a standardized test diet in 1,002 individuals (11). Dietary fermentable fiber is metabolized by gut bacteria and converted into short-chain fatty acids (SCFAs), which may contribute to host health, suggesting that differences in host gut bacterial composition may be directly related to differences in health outcomes. For example, an intervention study in Belgium in which individuals with obesity were administered inulin for 3 months, found higher pre-intervention levels of *Akkermansia* and *Butyricoccus* in the gut of those whose obesity improved after inulin supplementation (responders) (12). Additionally, an intervention study investigating the effect of barley on postprandial blood glucose levels reported a higher ratio of *Prevotella*/*Bacteroides* in the responder group, whose glucose tolerance improved after consuming barley bread, than in those whose glucose tolerance did not improve after consuming barley bread (non-responders) (13). In our previous study, which comprised Japanese participants older than 40 years, *Faecalibacterium* and *Subdoligranulum*, which are typical butyrate-producing bacteria, were found to be abundant in the intestines of the group that consumed more barley and did not suffer from dyslipidemia or hypertension (14, 15). In addition to dietary fiber, various foods have been reported to influence the gut bacteria. For example, fermented foods containing lactic acid bacteria, such as cultured dairy products and yogurt, are sources of ingestible microorganisms that may beneficially regulate gut health and treat or prevent inflammatory bowel disease (16). Another traditional Japanese food, natto, is made from soybeans fermented by *Bacillus subtilis*, a bacterium that reaches the intestinal tract because of its resistance to acid, bile, and digestive enzymes and has been reported to regulate the intestinal microbiota and be associated with lower blood lipid levels (17). The association between barley and obesity has the potential to be influenced by the gut microbiota and the consumption of other foods that affect the gut microbiota. In this study, 185 Japanese participants aged 20 or older were examined to profile the gut microbiota of those who consumed high amounts of barley and were not obese (responders) and to assess the association between fermented food intake and the presence or absence of obesity among the responders.

## 2 Materials and methods

### 2.1 Study design and implementation

This study was based on “The cohort study on barley and the intestinal environment (UMIN000033479),” which aimed to evaluate the association between barley and intestinal bacteria among employees of a company producing barley products. The original study was conducted in accordance with the principles of the Declaration of Helsinki, and sampling was conducted between August 2018 and March 2020.

**Figure 1:**
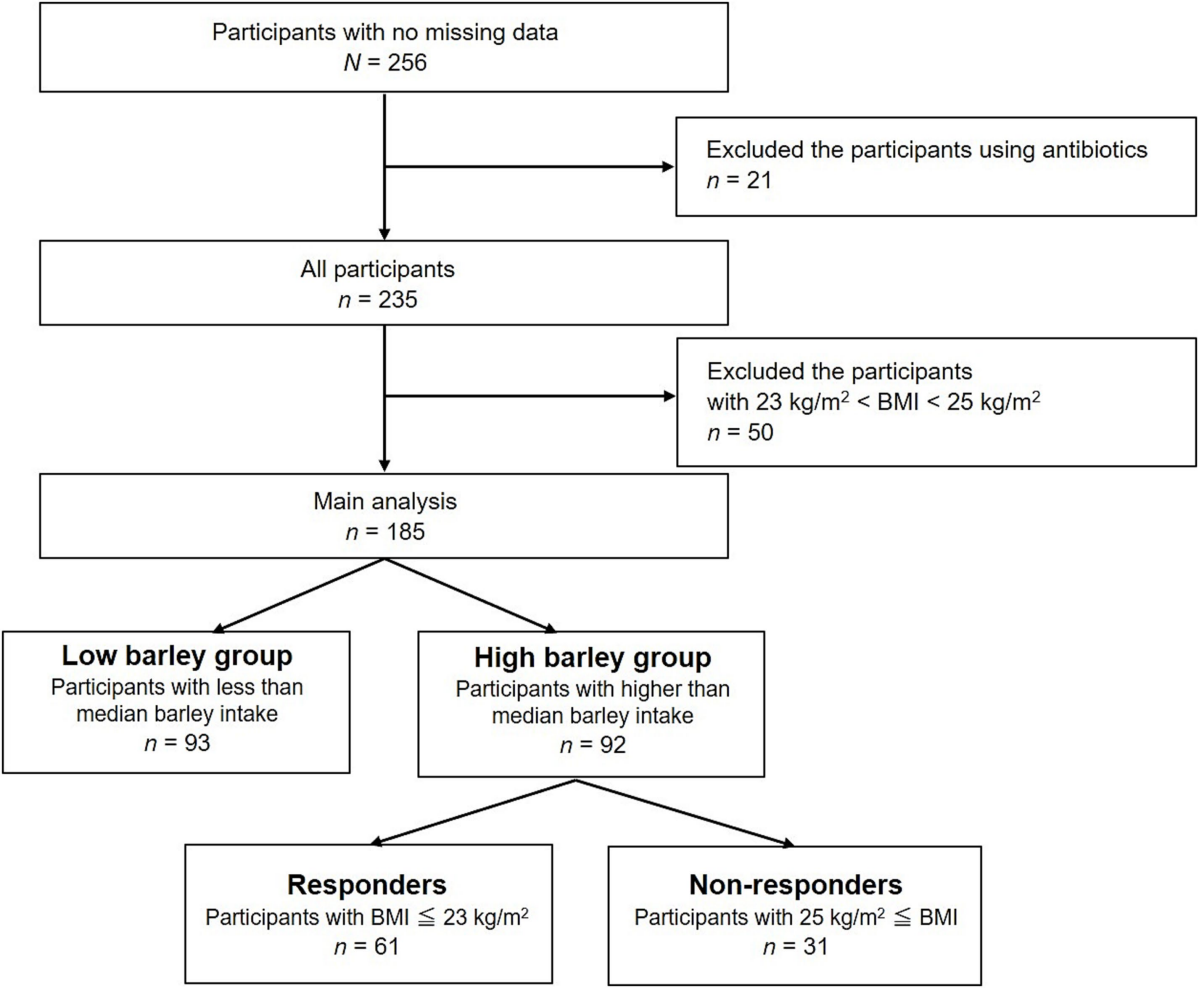
Flow chart of the recruitment and selection of participants.

A flowchart illustrating participant selection is shown in Figure 1. Of the consenting participants, 256 provided complete data. Twenty-one participants undergoing antibiotic treatment, potentially altering the gut microbiota, and 50 participants with a BMI in the borderline obesity range, 23 kg/m^2^ < BMI < 25 kg/m^2^, were excluded, resulting in a primary analysis of 185 participants.

All participants submitted health certificates for basic information such as weight and blood test results. Daily energy intake (kcal/day) was assessed using the brief self-administered diet history questionnaire (BDHQ, Gender Medical Research Inc., Tokyo, Japan), a simplified version of the diet history questionnaire (DHQ) developed to estimate dietary intake in accordance with Japanese dietary culture and habits. Daily barley intake (g/d) was calculated based on bowl size (200 g large, 160 g medium, 140 g small, and 100 g for children), the percentage of barley mixed with white rice (none, 5, 10, 15, 30, and 50%), daily intake frequency (times), and monthly intake frequency (none, 0.5, 1, 4, 8, 16 times, or more/month). Daily barley intake (g/1000 kcal) was calculated from total daily energy (kcal/d) and barley intake (g/d). Participants were stratified into the “low barley group” (*n* = 93) and “high barley group” (*n* = 92) based on the median barley intake (3.494 g/1000 kcal) of the 185 analyzed participants. The primary analysis focused on the high barley group, which was further stratified into 61 responders with BMI < 23 kg/m^2^ and 31 non-responders with BMI > 25 kg/m^2^.

### 2.2 16S rRNA gene amplicon sequencing and bioinformatics analysis

Fecal samples were collected from the participants at home using containers containing guanidine thiocyanate (GuSCN) solution (TechnoSuruga Laboratory, Shizuoka, Japan). These samples were stored at room temperature, and DNA extraction was conducted within 5 days following established protocols (18). Briefly, fecal samples in 0.2 mL of GuSCN solution were homogenized with 0.3 mL of lysis buffer (No. 10, Kurabo Industries Ltd., Osaka, Japan), 0.5 g of 0.1 mm glass beads (WakenBtech Co., Ltd., Tokyo, Japan), and 0.2 mL of GuSCN solution using a Cell Destroyer PS1000 (Bio Medical Science, Tokyo, Japan) at 4,260 rpm for 50 s at room temperature. The mixture was then centrifuged at 13,000 × g for 5 min at room temperature, and DNA was extracted from the supernatant using a GenePrep Star PI-80X instrument (Kurabo Industries, Osaka, Japan). DNA concentration was quantified using a NanoDrop Spectrophotometer ND-1000 (Thermo Fisher Scientific, Waltham, MA, United States), and the extracted DNA was stored at −30°C. Amplicon sequencing of the V3–V4 region of the 16S rRNA gene was performed as previously described (18). Barcoded amplicons were generated using the primers: forward, 5’-TCGTCGCAGCAGCAGATGTGTAGTAAGCGACAGCCTACGGNGGCWGCAG-3′; and reverse, 5’-GTCTCGTGGCTCGAGATGTATAAGACGACTACHVGGTATCTAATCC-3′. DNA libraries were prepared using the Illumina MiSeq platform (Nextera XT Index Kit v2 Set A; Illumina, San Diego, CA, USA). Library concentrations were determined using the QuantiFluor dsDNA System (Promega Co., Madison, WI, United States), and 16S rRNA gene sequencing was conducted using the Illumina MiSeq platform (Illumina). In instances where detailed methods were not available, the manufacturer’s recommendations were followed.

Sequence analysis was conducted using the Quantitative Insights into Microbial Ecology (QIIME) software package (version 1.9.1) with the QIIME Analysis Automating Script (Auto-q) (19). Paired-end read trimming for operational taxonomic unit (OTU) selection was carried out automatically, and OTUs were selected based on sequence similarity (> 97%) through open reference OTU picking using UCLUST software against SILVA v128 reference sequences (20, 21). Taxonomic classifications (phylum, order, family, and genus) and relative abundances were determined. Ten thousand reads were randomly selected for statistical analysis.

### 2.3 Statistical analysis

Unless specified otherwise, all statistical analyses were conducted utilizing R version 3.6.0.

#### 2.3.1 Taxonomical analysis

We calculated alpha diversity metrics, including Chao1, Shannon, and Simpson, to assess within-subject bacterial diversity using the richness function available in the phyloseq R package. Bray-Curtis distance was used to calculate *β*-diversity, reflecting bacterial diversity among participants. Principal coordinate analysis (PCoA) was performed by integrating the two-dimensional data of all genus-level enterobacteria using the vegdist and quasi-eyelid functions from the vegan R package, along with the dudi.pco function from the ade4 R package.

#### 2.3.2 Comparative analysis among participant groups

Age, BMI, blood pressure, fasting blood glucose, hemoglobin (Hb) A1c, triglycerides, low-density lipoprotein cholesterol, high-density lipoprotein cholesterol, and dietary intake were compared between non-responders and responders using the Student’s *t*-test. Three group comparisons of the responder, non-responder, and low barley groups were performed using one-way analysis of variance (ANOVA). The sex distribution between the two groups was assessed using the chi-square test. Then, the Mann–Whitney *U* test was employed to compare *α*-diversity, PCo1, and PCo2, as well as the relative abundance of enterobacteria between the two groups. The relative abundance of bacteria among the three groups was analyzed using the Kruskal-Wallis test. The analysis focused on 64 genera with an average relative abundance of 0.1% or higher among study participants. Statistical significance was set at *p* < 0.05.

#### 2.3.3 Mitigation of confounding effects

We conducted a logistic regression analysis to assess the relationship between responders and their characteristic bacteria. Responders (coded as 1 = responders, 0 = non-responders) were the objective variable, while the relative abundance of each bacteria and various adjusting covariates (sex [male = 0, female = 1]; age; dyslipidemia risk [absent = 0, borderline = 1, presence = 2]; hypertension risk [absent = 0, borderline = 1, presence = 2]; and T2DM risk [absent = 0, borderline = 1, presence = 2]) were considered as explanatory variables. Variance inflation factors (VIFs) were examined using the vif function in the car R package, and all VIFs were below 2, warranting their inclusion in the model.

#### 2.3.4 Main effect of responder status on gut bacteria

To measure the main effects of responders and confounding factors on gut bacteria specific to responders, multi-way ANOVA was conducted. The dependent variable was the relative abundance of each bacterium specific to responders, while the independent variables included responder status (non-responders = 0 responders = 1); sex (male = 0 female = 1); age; dyslipidemia risk (absence = 0, borderline = 1, presence = 2); hypertension risk (absence = 0, borderline = 1, presence = 2); and T2DM risk (absence = 0, borderline = 1, presence = 2). Interaction analyses were added to observe the potential interactions when significant main effects were found for more than one independent variable.

#### 2.3.5 Analysis of the interaction between barley and natto

Initially, the participants were stratified based on their barley and natto intake. The main study participants (*n* = 185) were classified into four groups based on the median intake levels of barley (3.494 g/1000 kcal) and natto (2.2 g/1000 kcal) (Supplementary Figure S1). The distribution of individuals classified as healthy (BMI < 23 kg/m^2^) or obese (BMI > 25 kg/m^2^) across the four groups was assessed using the chi-square test. Subsequently, to evaluate the interaction between barley and natto intake, the relative abundances of intestinal bacteria characteristic of responders within these four groups were compared using the Kruskal-Wallis test. Finally, to address confounding factors, the high-barley high-natto group was designated as the objective variable (high-barley high-natto group = 1, low-barley high-natto group = 0), and a logistic regression analysis was performed. The explanatory variable in this model was the relative abundance of each bacterium, and the adjusted variables included sex; age; dyslipidemia risk (absent = 0, borderline = 1, and presence = 2); hypertension risk (absent = 0, borderline = 1, presence = 2); and T2DM risk (absent = 0, borderline = 1, presence = 2).

#### 2.3.6 Machine learning approaches for model development

We developed a classification model using a random forest, a widely used supervised learning algorithm, to predict the response of barley to obesity. This algorithm was selected due to its robustness in handling high-dimensional biological data and its ability to prevent overfitting. All the random forest classification models were constructed using the RandomForest and caret R packages. The dataset of 92 participants from the high barley intake group was divided into 60% (*n* = 56) and 40% (*n* = 36) training and test sets, respectively, while maintaining a fair ratio of responders to non-responders. The classification model was developed using a 10-fold cross-validation with 10 iterations. The oneSE rule was applied to prevent overfitting and the mtry parameter was adjusted using the caret R package. The ntree was set to 500 and other parameters were initialized with the default values recommended by the RandomForest R package. A receiver operating characteristic (ROC) analysis was conducted using the ROCR R package to evaluate the performance of the classification model. The objective variable for these classification models was the presence or absence of obesity (Responder or Non-responder). In these classification models, we incorporate three types of explanatory variables: 1. the relative abundance (%) of 64 genera with an average relative abundance greater than 0.1%; 2. relative abundance (%) of 64 genera and intake of five fermented foods (yogurt, yogurt drink, natto, pickled bran, kimchi) (g/1000 kcal); and 3. intake of five fermented foods (g/1000 kcal).

## 3 Results

### 3.1 Participant demographics and characteristics

A total of 185 participants were included in the analysis, with a mean age of 39 ± 12 years (range 20–64 years) and comprising 134 (72%) were men. The average barley intake was 5.1 ± 5.2 g/1000 kcal (ranging from 0 to 50.8 g/day), with a median intake of 3.494 g/1000 kcal. Based on this median barley intake, the participants were stratified into low (*n* = 93) and high barley intake groups (*n* = 92). Within the high barley intake group, individuals with a BMI < 23 kg/m^2^ were categorized as responders, whereas those with a BMI > 25 kg/m^2^ were categorized as non-responders, resulting in 61 responders and 31 non-responders (Figure 1). Table 1 presents detailed characteristics of the participants. The responders were notably younger than the non-responders and exhibited a higher proportion of females. Additionally, the responders had lower BMIs, smaller waist circumferences, and generally more favorable health profiles. This indicates that the barley responders with obesity may represent a subgroup at lower risk not only for obesity but also for other lifestyle-related diseases. Furthermore, the dietary analysis revealed that the responders had significantly lower daily calorie intake and macronutrient consumption than the non-responders. These findings suggest that variations in energy intake can partially account for differences in BMI. Notably, intake of fermented foods, natto, which may impact gut microbiota composition, was significantly higher in responders (7.6 ± 9.4 g/1000 kcal (crude 0–50 g/day)) compared to non-responders (3.9 ± 5.1 g/1000 kcal (crude 0–36 g/day)).

**Table 1:** Participant demographics and characteristics.

		Responders (*n* = 61)	Non-responders (*n* = 31)	*p*-value^1^	Low barley group (*n* = 93)	*p*-value^3^
Age		37 ± 12	42 ± 13	0.04	40 ± 12	0.09
Male (*n*)		34 (56%)	28 (90%)	< 0.01 ^2^	72 (77%)	< 0.01 ^2^
Barley intake (g/1000 kcal)		8.0 ± 4.5	10.3 ± 5.6	0.04	1.4 ± 1.2	< 0.01
Medical checkup						
BMI (kg/m^2^)		20.1 ± 1.7	28.6 ± 3.7	< 0.01	23.2 ± 4.1	< 0.01
Waist (cm)		72.2 ± 5.7	93.6 ± 11.2	< 0.01	82.2 ± 11.2	< 0.01
SYS (mm/Hg)		113 ± 16	127 ± 16	< 0.01	119 ± 14	< 0.01
DIA (mm/Hg)		70 ± 12	81 ± 12	< 0.01	74 ± 11	< 0.01
Glucose (mg/dL)		85 ± 8	96 ± 17	< 0.01	92 ± 15	< 0.01
Hemoglobin A1c (%)		5.3 ± 0.2	5.7 ± 0.6	< 0.01	5.5 ± 0.6	< 0.01
Triglycerides (mg/dL)		71 ± 32	149 ± 75	< 0.01	109 ± 72	< 0.01
HDL-cholesterol (mg/dL)		66 ± 14	50 ± 11	< 0.01	60 ± 14	< 0.01
LDL-cholesterol (mg/dL)		100 ± 27	129 ± 28	< 0.01	115 ± 29	< 0.01
Nutrients						
Energy (kcal/d)		1,613 ± 438	2000 ± 627	< 0.01	1763 ± 569	<0.01
Protein (g/d)		56 ± 16	65 ± 22	0.04	60 ± 20	0.15
Fat (g/d)		47 ± 14	54 ± 23	0.049	51 ± 19	0.14
Carbohydrate (g/d)		211 ± 66	265 ± 98	< 0.01	223 ± 81	<0.01
Fermentation foods (g/1000 kcal)						
Yogurt		11.3 ± 20.0	10.8 ± 17.4	0.91	15.3 ± 34.8	0.61
Yogurt drink		11.9 ± 21.5	5.9 ± 10.7	0.14	10.2 ± 20.0	0.37
Natto		7.6 ± 9.4	3.9 ± 5.1	0.04	4.2 ± 5.7	<0.01
Pickled bran		2.0 ± 6.8	4.7 ± 16.2	0.26	2.7 ± 7.6	<0.01
Kimchi		1.3 ± 2.1	2.0 ± 4.4	0.30	1.3 ± 2.6	0.48

BMI, body mass index; SYS, Systolic blood pressure; DIA, diastolic blood pressure; HDL, high-density lipoprotein; LDL, low-density lipoprotein. ^1^Responders and non-responders were compared using the Student’s t-test. ^2^Responders and non-responders were compared using Pearson’s chi-squared test. ^3^Three group comparisons of the responder, non-responder, and low barley groups were performed using the one-way analysis of variance.

### 3.2 Barley responders gut microbiota diversity

**Figure 2:**
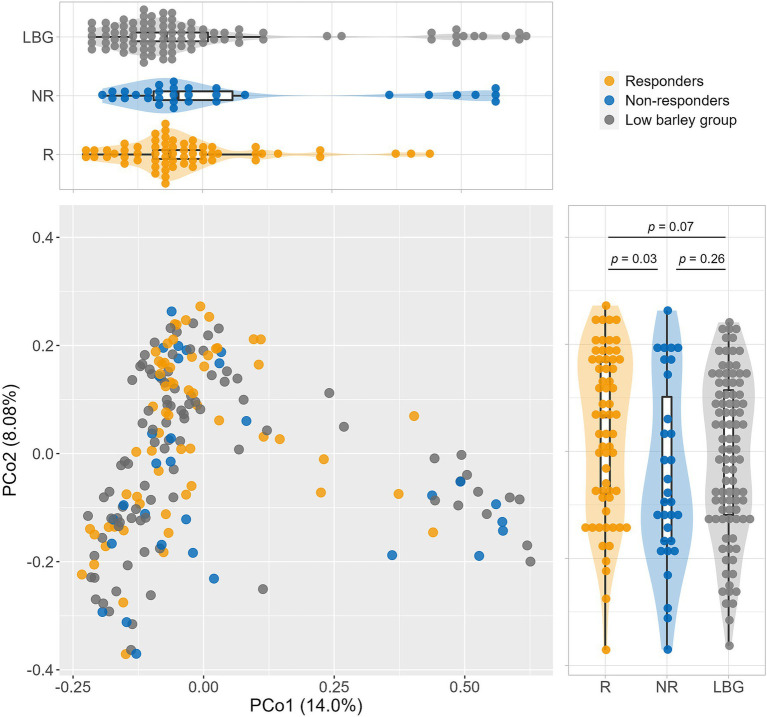
Comparison of the gut microbiome composition. Principal coordinate analysis (PCoA) of gut microbiome based on 266 genera abundance.

**Figure 3:**
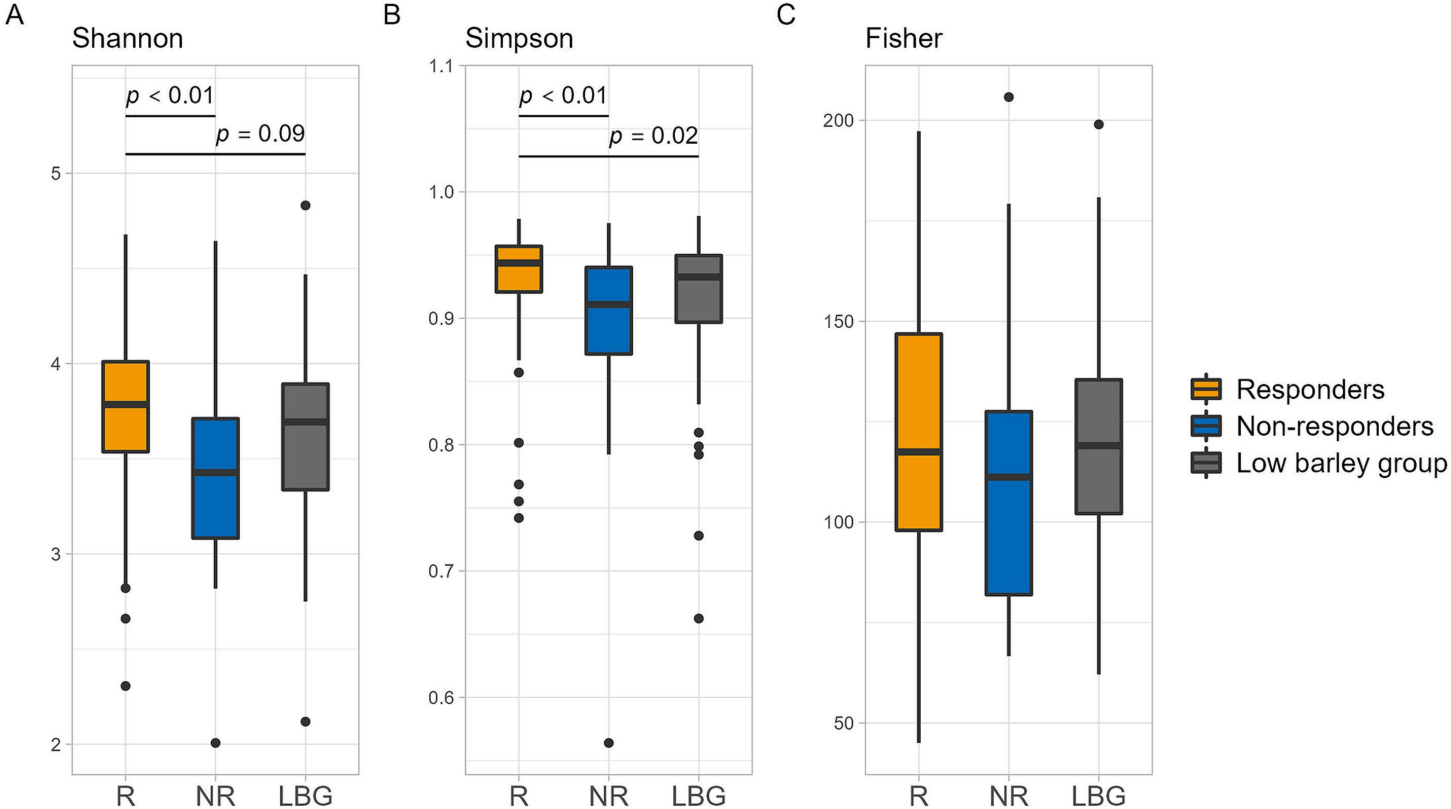
*α* diversity of intestinal bacteria in each group. **(A)** Shannon, **(B)** Simpson, and **(C)** Fisher. *p* values are calculated by the Mann–Whitney *U* test.

Analysis of *β*-diversity via PCoA revealed significant differences between responders and non-responders at PCo2, with no significant difference observed between the non-responder and low barley intake groups. However, the responder group exhibited distinct tendencies compared with both the non-responder and low barley intake groups. Moreover, the combined contribution of PCo1 and PCo2 accounted for 22% of the variation in gut microbiota among barley responders; this result may not fully capture the overall structure. However, the gut microbiota of responders may be distinctive (Figure 2). In terms of *α*-diversity, among several indices, responders showed significantly higher values in Shannon and Simpson indices compared to non-responders, indicating a potentially more favorable gut environment, supporting a more diversity gut microbial community in responders (Figure 3).

### 3.3 Distinctive gut bacteria in barley responders

To identify the gut bacteria characteristics of responders, we compared the relative abundances of 64 genera with a mean relative abundance of ≥0.1% between responders and non-responders using the Mann–Whitney *U* tests. Consequently, the abundances of nine genera, including *Bifidobacterium* (*p* = 0.02), Christensenellaceae R7 group (*p* = 0.011), Lachnospiraceae ND3007 group (*p* = 0.048), Lachnospiraceae NK4A136 group (*p* = 0.048), *Butyricicoccus* (*p* = 0.002), *Ruminiclostridium* 5 (*p* = 0.007), *Subdoligranulum* (*p* = 0.02), Ruminococcaceae uncultured (*p* = 0.02), and *Megamonas* (*p* = 0.02), were significantly different between responders and non-responders, with all except *Megamonas* being enriched in responders (Table 2).

**Table 2:** Characteristics of the microbiome in three study groups.

	Responders			Non-responders				Low barley group			
	Median	1st Qu	3rd Qu	Median	1st Qu	3rd Qu	*p-*value ^1^	Median	1st Qu	3rd Qu	*p-*value ^2^
*Bifidobacterium*	5.21	1.91	10.09	2.18	0.55	6.005	0.02	2.7	1.12	7.13	< 0.01
Christensenellaceae R7 group	0	0	0.08	0	0	0	0.011	0	0	0.01	0.03
Lachnospiraceae ND3007 group	0.08	0	0.17	0.02	0	0.09	0.048	0.02	0	0.12	0.11
Lachnospiraceae NK4A136 group	0.33	0.03	0.73	0.10	0.01	0.42	0.048	0.21	0.02	0.61	0.13
*Butyricicoccus*	0.66	0.47	0.88	0.46	0.20	0.64	< 0.01	0.47	0.23	0.7	< 0.01
*Ruminiclostridium* 5	0.14	0.05	0.29	0.04	0.02	0.16	< 0.01	0.11	0.05	0.29	0.02
*Subdoligranulum*	1.90	0.79	3.39	0.34	0.01	1.51	0.012	0.77	0.01	2.08	< 0.01
Ruminococcaceae uncultured	0.20	0.08	0.65	0.07	0.02	0.31	0.02	0.2	0.06	0.51	0.06
*Megamonas*	0	0	0	0	0	9.49	0.02	0	0	2.65	< 0.01

^1Responders and non-responders were compared using the Mann–Whitney U-test. 2Three group comparisons of the responder, non-responder, and low barley groups were performed using the Kruskal-Wallis rank sum test.^

**Figure 4:**
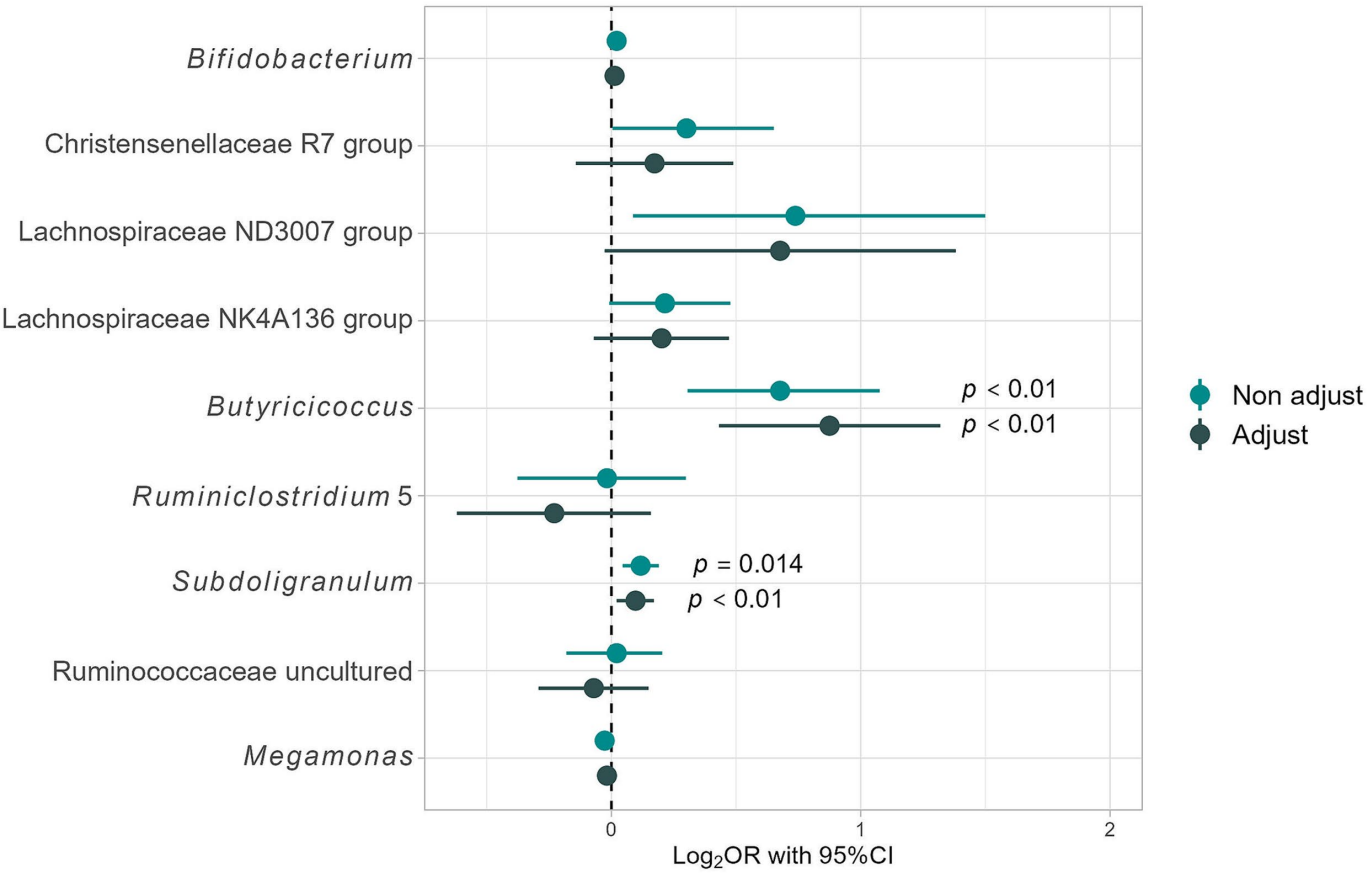
Differences in intestinal bacterial composition based on logistic regression analysis. Results for Non-adjust: the target variables are “Responders” (1) and “Non-responders” (0), and the dependent variables are the presence ratio (%) of each gut bacterium. Additionally, adjust results consider adjustment variables such as age; sex; dyslipidemia risk (0, 1, 2), hypertension risk (0, 1, 2), and T2DM risk (0, 1, 2), providing a comprehensive analysis of the impact of these factors on gut microbiome composition.

The responders in this study differed significantly from the non-responders in terms of age, sex ratio, and other health factors besides BMI. As a result, adjustments for these confounding factors were required to assess the differences in gut bacterial abundance. Therefore, a logistic regression analysis was conducted for the nine aforementioned genera of gut bacteria; the results are shown in Figure 4. The “non-adjust” values represent the results of explaining the dependent variable (non-responder, responder = 1, 0) using each gut bacterium as an independent variable. The “adjust” values represent the justification of the dependent variable with each gut bacterium as an independent variable and adjusting for confounding factors [age; sex; diabetes risk (0, 1, 2); hypertension risk (0, 1, 2); and dyslipidemia risk (0, 1, 2)]. After adjusting for confounding factors, no significant differences were found for most gut bacteria; however, Lachnospiraceae ND3007 group [Odds ratio (OR): 4.75, 95% confidence interval (95% CI: 0.94–24.06; *p* = 0.06)] tended to be enriched in responders, while *Butyricicoccus* (7.50; 2.70–20.85; *p* < 0.01) and *Subdoligranulum* (1.25; 1.05–1.48; *p* = 0.01) were significantly enriched in responders (Figure 4). Therefore, *Butyricicoccus* and *Subdoligranulum* are inferred to be key gut bacteria for assessing the presence of obesity in individuals with high barley consumption.

For sensitivity analysis, we measured the main effect of responder status on gut bacteria characteristic of responders using multi-way ANOVA. Significant main effects of responder status were observed for *Butyriciccocus* and *Subdoligranulum* (*Butyriciccocus*: *F* = 14.18, *p* < 0.01; *Subdoligranulum*: *F* = 12.23, *p* < 0.01) (Supplementary Table S1). For *Subdoligranulum*, a significant main effect of age was found, in addition to responder status. Therefore, an interaction analysis between these two factors was conducted; however, no significant interaction was found (*F* = 0.70, *p* = 0.40). These results suggest that responder status independently affects these two gut bacteria. The detailed results are presented in Supplementary Table S1.

### 3.4 Intestinal bacteria degrading barley *β*-glucan

**Figure 5:**
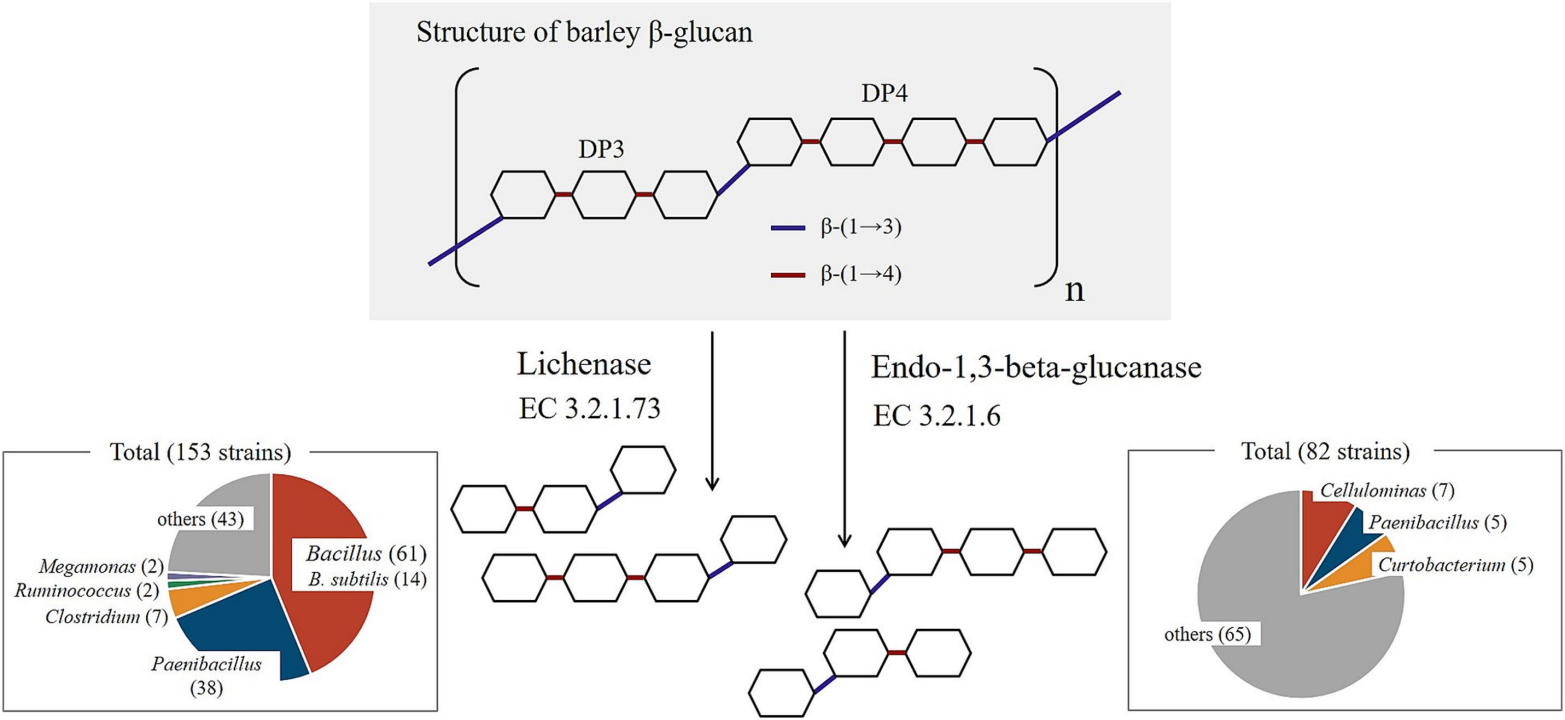
Enzymes that specifically degrade barley β-glucan and bacteria that have these enzymes.

Barley contains β-glucan in the form of β-(1 → 3)-(1 → 4)-glucan (22). The enzymes that specifically break down this dietary fiber are lichenase (EC 3.2.1.73) and endo-1,3-beta-glucanase (EC 3.2.1.6) (23) (Figure 5). These enzymes are present in many plants of the Poaceae family and yeast. Therefore, we evaluated bacteria that possess these two enzymes based on the Kyoto Encyclopedia of Genes and Genomes (KEGG) database. Among the 8,187 registered bacterial strains, 153 strains, represented by *Bacillus*, possessed lichenase and 82 strains possessed endo-1,3-beta-glucanase (as of February 10, 2024) (Figure 5, Supplementary Table S2). Many of these bacteria are soil bacteria, and few are common intestinal bacteria. Additionally, one strain of *Subdoligranulum*, a characteristic bacterium of responders, is registered in the KEGG database (*Subdoligranulum* var*iabile* DSM 15176). However, this strain was not found to possess lichenase or endo-1,3-beta-glucanase. Furthermore, *Butyricicoccus* was not registered in the KEGG database.

### 3.5 The quantity of *Bacillus* in the intestine and its relationship with barley and natto

**Figure 6:**
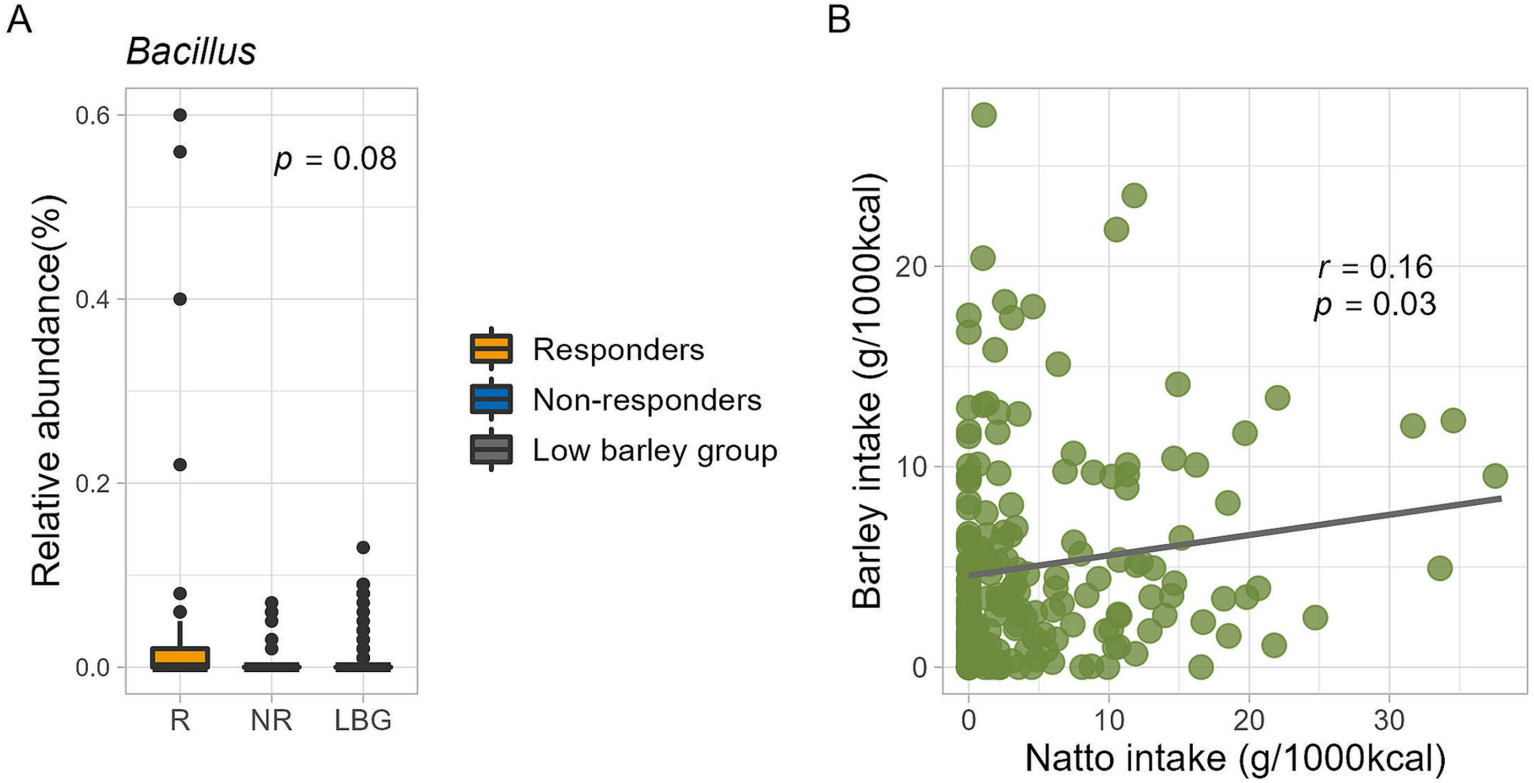
**(A)** *Bacillus subtilis* abundance in this study. The *p*-value is calculated by the Kruskal-Wallis test. **(B)** Relationship between barley and natto intake for all participants (*n* = 185). The correlation coefficient and *p*-value are calculated by Spearman’s rank correlation coefficient.

*Bacillus*, characterized by its frequent occurrence of lichenase that specifically breaks down barley *β*-glucan, is not commonly found in the intestinal flora. Conversely, *B. subtilis*, a bacterium utilized in the fermentation of natto, a traditional Japanese fermented food, is known to survive the intestinal harsh conditions because of its resistance to gastric and bile acids (24, 25). In the present study, the overall prevalence of *Bacillus* was 24%, with a mean relative abundance of 0.02%. Moreover, *B. subtilis* was found in 34% (*n* = 21) of the respondents and 19% (*n* = 6) of the non-respondents, indicating a higher prevalence among the respondents, although the difference was not statistically significant. Additionally, the intestinal abundance of *B. subtilis* was notably higher (*p* = 0.08, Kruskal-Wallis test) in respondents than that in non-respondents (Figure 6A).

**Figure 7:**
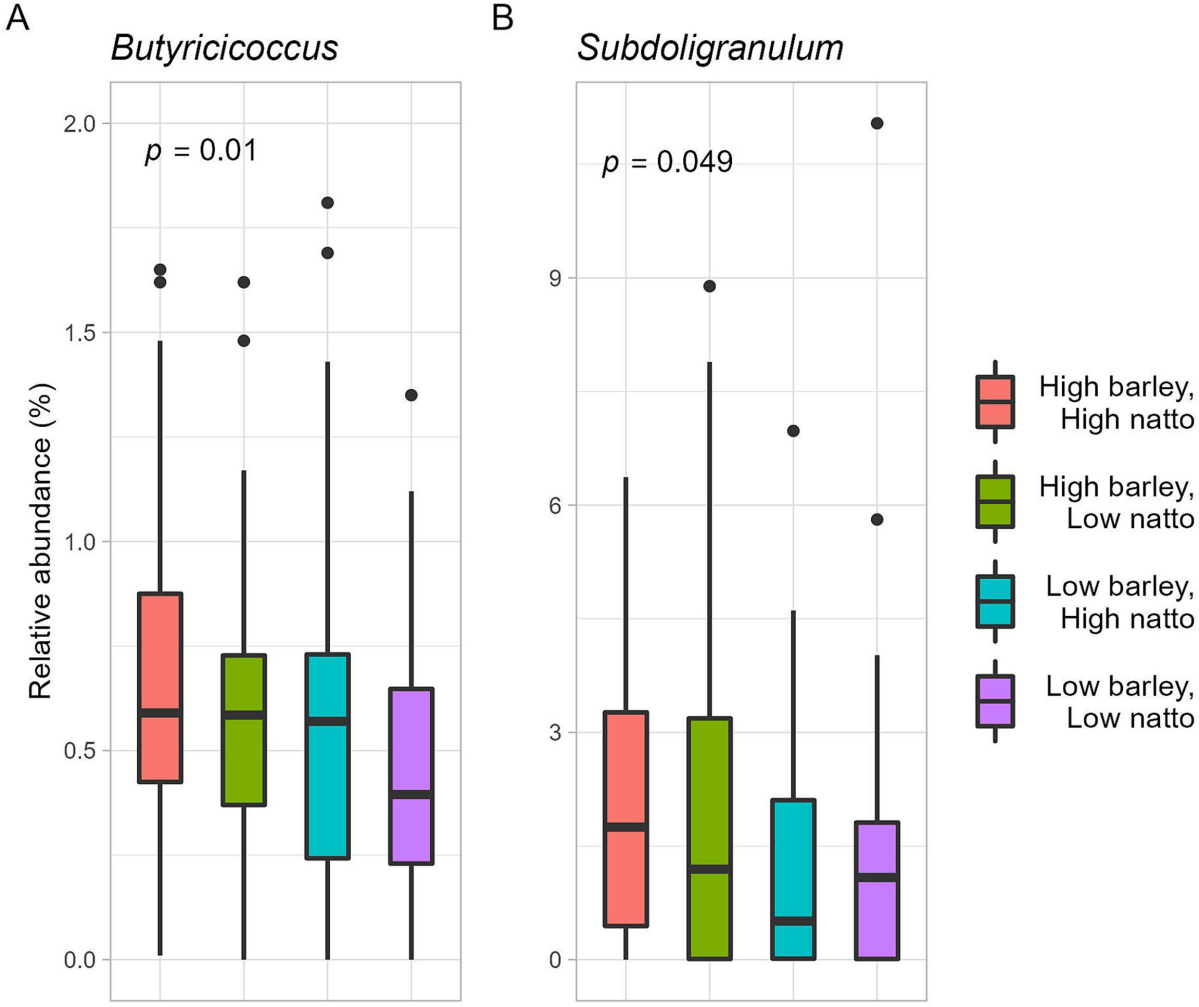
Relative abundance of responder characteristic bacteria in relation to mutual intake of barley and natto. The *p*-value is calculated by the Kruskal-Wallis test.

**Figure 8:**
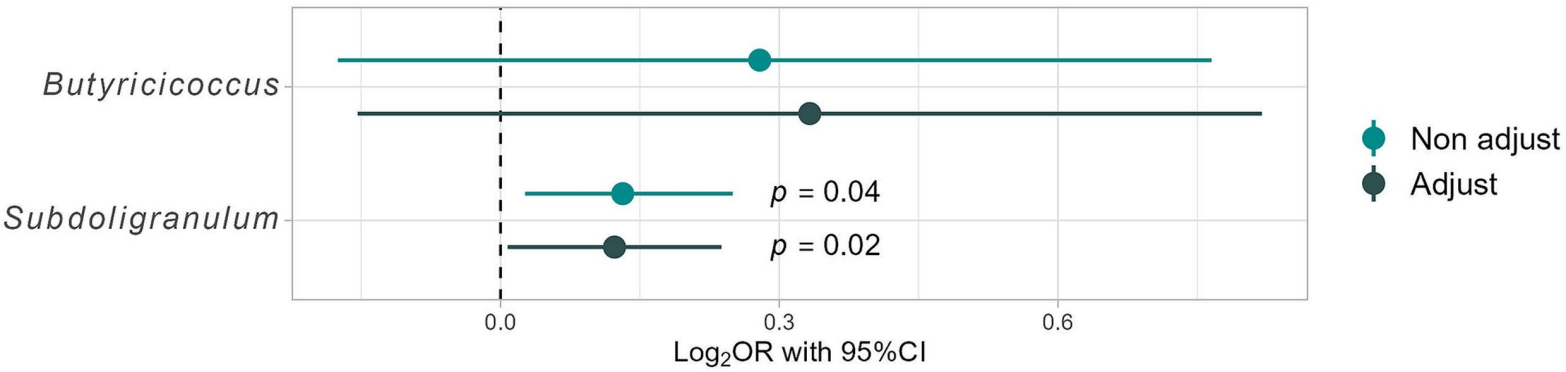
Differences in responder characteristic bacteria in the high barley-high natto group by logistic regression analysis. The model is constructed under the following conditions. Results for Non-adjust: the target variables are “High barley-high natto group” (1) and “Low barley-high natto group” (0), and the dependent variables are the presence ratio (%) of each gut bacterium. Additionally, adjust results considered adjustment variables, such as age; sex; dyslipidemia risk (0, 1, 2), hypertension risk (0, 1, 2), and T2DM risk (0, 1, 2).

A subtle positive correlation was observed between barley and natto consumption (all participants, *r* = 0.16, *p* = 0.03, Pearson product–moment correlation coefficient) (Figure 6B), suggesting that these items are integral to the traditional Japanese diet and are often consumed concurrently. Further analysis, the participants were stratified into four groups based on the median barley and natto intake (Supplementary Figure S1A). No disparity in obesity incidence (*p* = 0.42 by Chi-squared test) was found between the high and low barley-high natto groups (Supplementary Figure S1B). Examination of responder-characteristic bacterial abundance across the groups revealed that *Butyricicoccus* and *Subdoligranulum* were notably prevalent in the high barley-high natto group (*Butyricicoccus*, *p* = 0.01; *Subdoligranulum*, *p* = 0.049; Kruskal-Wallis test), suggesting an interactive relationship between barley and natto (Figure 7). Logistic regression analysis of the two genera of intestinal bacteria characteristic of responders revealed that *Subdoligranulum* was significantly more abundant in the high-barley high-natto group after adjusting for confounders (Figure 8).

### 3.6 Prediction of barley responders using random forest

**Figure 9:**
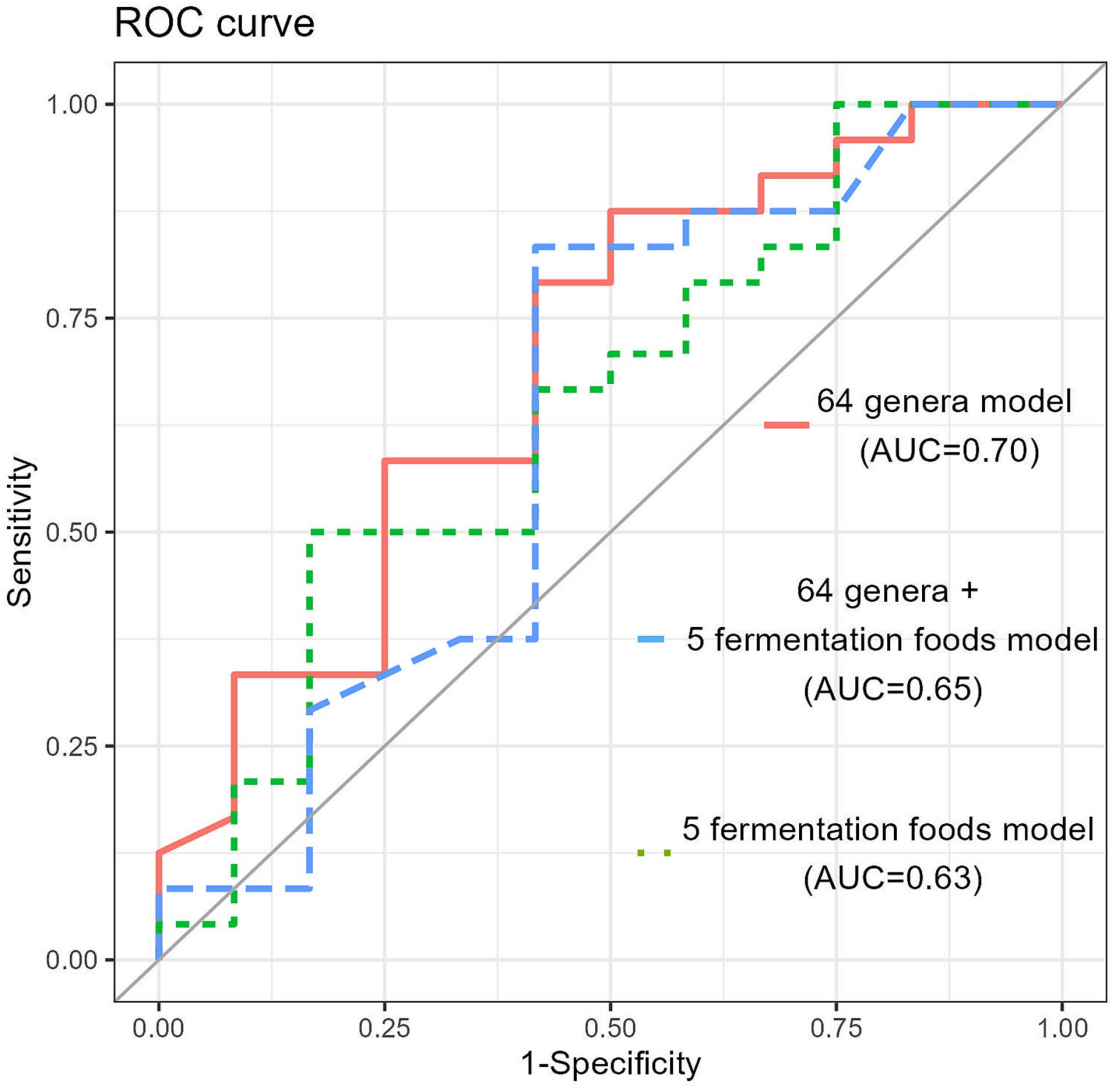
The random forest classification model generated based on 64 genera in the training data set. The receiver operating characteristic (ROC) curves and area under the curve (AUC) of the microbiome for discrimination between responders and non-responders.

A classification model was developed, with responder or non-responder as the target variable and 64 genera of gut bacteria as predictors, yielding an area under the curve (AUC) of 0.70 (cutoff, 0.572; sensitivity, 87.5%; and specificity, 50.0%). Expanding on the previous model, we included the intake of five fermented food items as additional predictors in the new classification model. The most accurate model achieved an AUC of 0.646 (sensitivity, 50.0%; specificity, 83.3%; and cut-off, 0.712). Furthermore, when only the intake of the five fermented food items was considered as the target variable, the AUC was 0.630 in the classification model (Figure 9).

## 4 Discussion

In this study, we hypothesized that variations in the impact of barley intake on host obesity may be attributed to differences in individuals’ gut bacteria. We defined individuals with high barley intake but not obesity as responders and those with high barley intake and obesity as non-responders, and investigated differences in their gut bacterial composition. Consequently, the responders showed a higher abundance of *Butyricicoccus* and *Subdoligranulum* than the non-responders, suggesting that these two genera define the barley responders and may influence the relationship between barley intake and obesity.

In recent years, numerous studies have highlighted the pivotal role of gut microbiota in modulating obesity. For instance, investigations have revealed a marked discrepancy in the abundance of *Bacteroides thetaiotaomicron* between healthy and obese individuals. In a cohort comprising 95 young obese individuals (BMI, 37.03 ± 4.69 kg/m^2^) alongside 105 age-and sex-matched controls (BMI, 20.24 ± 1.26 kg/m^2^), *B. thetaiotaomicron* levels were found to be notably higher in the former group. Moreover, oral administration of *B. thetaiotaomicron* to diet-induced obese mice significantly attenuated weight gain (26). Similarly, a cross-sectional analysis of 217 Japanese participants revealed an inverse correlation between *Blautia* abundance, incidence of obesity, and T2DM. Notably, oral supplementation with *B. wexlerae* led to a remarkable suppression of obesity and abnormal glucose metabolism in diet-induced obese mice (27), which underscores the pivotal role of *Blautia* in mitigating obesity by modulating the gut microbiota composition and enhancing the production of SCFAs via interactions with commensal bacteria, such as *Butyricicoccus*. SCFAs synthesized by gut bacteria contribute to 10% of the daily energy requirements (28) and serve as regulatory molecules that are beneficial for host energy homeostasis (29). Furthermore, butyrate generated by butyrate-producing bacteria, such as *Feacalibacterium prausnitzii*, may also exert anti-obesity effects by stimulating GLP-1 secretion from colon L cells via fatty acid receptor (FFAR2)-mediated signaling and mitigating insulin resistance (30, 31). Notably, reduced levels of butyrate in fecal samples have been observed in obese individuals compared with those with normal weight (32). Hence, the prevalence of SCFA-producing bacteria may be advantageous for combating obesity. The observation that *Butyricicoccus* and *Subdoligranulum* were abundant in the intestines of barley responders suggests that butyrate synthesis could be enhanced in the gut of these individuals.

However, SCFA-producing bacteria are unlikely to directly break down dietary fibers to produce SCFAs, as metabolic relays among various gut bacteria are thought to be necessary for SCFA generation. Dietary fiber is initially degraded by saccharolytic bacteria, such as *B. subtilis*, in the gut into simpler compounds, which are then converted into lactic acid, acetic acid, and other metabolites by saccharolytic bacteria, such as lactobacilli and *Bifidobacterium*. These metabolites are further metabolized by SCFA-producing bacteria into propionic and butyric acids, thereby benefiting host health. Previous studies have found that a synbiotic formulation containing *B. subtilis* and L-alanyl-L-glutamine increased the abundance of butyrate-producing bacteria and the butyrate concentration in the feces of 18 healthy men (33). Additionally, cross-feeding interactions between *Bifidobacterium*, which breaks down oligosaccharides and inulin, and other gut bacteria like *Faecalibacterium* and *Roseburia*, efficiently produce butyrate (34, 35).

Several intestinal bacteria capable of depolymerizing barley *β*-glucan have been reported. In a previous *in vitro* study, it was revealed that *Blautia producta* possesses genes associated with barley β-glucan hydrolysis. Other intestinal bacteria, including those from the *Lachnospira*, were reported to have proteins highly similar to the those encoded by these genes (36). Another *in vitro* study revealed that the addition of barley *β*-glucan to the medium enhanced the growth of 21 of the 49 strains of human gut bacteria belonging to 23 genera. Interestingly, while *Subdoligranulum* growth was not enhanced in the medium with added barley *β*-glucan, it significantly increased in media supplemented with low molecular weight barley *β*-glucan-derived DP3 and DP4, hydrolyzed by *Bacteroides ovatus* ATCC8483T. This suggests the potential for metabolic relay by specific gut bacteria even in barley β-glucan metabolism (37). Therefore, although β-glucan is degraded in the intestines of many humans, the varying growth abilities among species and strains of even the same genus of intestinal bacteria, coupled with the inability of these experiments to precisely mimic the human digestive environment, necessitate further investigated to elucidate the metabolism of barley β-glucan in the gastrointestinal tract. Furthermore, upon examination of the KEGG database, intestinal bacteria possessing enzymes capable of specifically degrading barley β-glucan, such as lichenase (EC 3.2.1.73) and endo-1,3-beta-glucanase (EC 3.2.1.6), were identified. Intestinal bacteria harboring lichenases were found in three genera: *Megamonas* (registered strains: owned strains = 2:2), *Clostridium* (66:7), and *Ruminococcus* (6:2), suggesting that most intestinal bacteria do not possess these enzymes. According to the KEGG database, bacteria possessing lichenase are predominantly soil bacteria, with *Bacillus* species being the most common, particularly *B. subtilis* (Figure 5).

*B. subtilis* is a Gram-positive, catalase-positive bacterium found widely in soil and plants, known for its role in fermenting soybeans to produce natto, a traditional Japanese delicacy. Upon reaching the upper part of the small intestine, *B. subtilis* exhibits metabolic activity by germinating into trophoblasts. Notably, studies have indicated transient colonization of intestinal epithelial cells (38–40). Metagenomic analysis of 787 Japanese individuals revealed a higher prevalence of *B. subtilis* in their intestines than in overseas populations, likely owing to natto consumption (41). In this study, a slightly positive association between natto and barley intake was observed across all participants (Figure 6B). Moreover, responders exhibited higher natto consumption than non-responders (Table 1) and tended to harbor more abundant *B. subtilis* in their intestines (Figure 6A). On the other hand, the genus *Bacillus* comprises different bacterial species, some of which are often observed in the human gut (42, 43). Therefore, a physicochemical association cannot be demonstrated between barley and *B. subtilis* based on the results observed in this study. However, the significant interaction between barley and natto in responders may provide a more comprehensive explanation for the association between barley consumption and obesity. Inferring from these results, *Bacillus* in responders may efficiently convert barley *β*-glucan to glycosides in the gastrointestinal tract, potentially mediating the metabolic activity of other intestinal bacteria and activating the production of SCFAs.

Natto has various health benefits. A previous animal investigation revealed that *B. subtilis* JLCC513, found in natto, diminished obesity in obese rats by enhancing intestinal microflora and intestinal barrier function (44). Additionally, the abundance of natto kinase in natto mitigates hypertension, thereby reducing the risk of cardiovascular disease (45–47). Given the frequent co-occurrence of hypertension and obesity (48), natto consumption may influence obesity. However, in our study, responders exhibited higher natto consumption compared to non-responders (OR: 1.01; 95% CI: 1.00–1.02; *p* = 0.004, results not shown), even after adjusting for confounding factors like the risk of lifestyle-related diseases. This suggests an interaction between barley and natto and indicates these foods may have synergistic effects on human health.

Furthermore, an investigation into the association between barley and natto intake and the two gut bacterial characteristics of responders revealed that both *Butyricicoccus* and *Subdoligranulum* were significantly more abundant in the high barley-high natto intake group, indicating an interaction effect between barley and natto (Figure 7). Although *Butyricicoccus* and *Subdoligranulum* are common butyrate-producing bacteria, studies on their physiological roles are limited. However, barley and natto intake may determine the abundance of these bacteria in the gut, thereby providing crucial insights into the biochemical understanding of the microbial metabolism of barley *β*-glucan and its impact on human health. Further analysis, even after adjusting for confounding factors, revealed that *Subdoligranulum* remained abundant in the high barley-high natto intake group, suggesting that *Subdoligranulum* may be a potent intestinal bacterium that defines the barley responder status (Figure 8). These results suggest that in barley responders, *B. subtilis*, a glycolytic bacterium, may create an environment in the digestive tract conducive to efficient energy conversion of barley dietary fiber by intestinal bacteria through the low molecular weight of barley β-glucan. Although the timing of barley and natto intake was not investigated in this study, both foods being traditional Japanese cuisines may imply concurrent consumption. Further studies are warranted to elucidate how the timing of barley and natto consumption affects intestinal bacteria and obesity.

In the construction of a classification model using random forest in an additional analysis, the model with 64 genera of gut bacteria as explanatory variables outperformed the model with fermented food items added as explanatory variables in classifying barley responders with higher accuracy. Although *B. subtilis* in responders is considered an important dietary factor contributing to barley degradation, this result suggests that responders are more strongly defined by their gut microbiota composition than that by dietary factors.

This study had some limitations. First, the BDHQ used to estimate dietary intake in this study is a more accessible version of the self-administered DHQ, which is known to have moderate validity and tends to underestimate energy intake (49). However, evaluations comparing this questionnaire with actual semi-weighted dietary records suggest that using energy-adjusted values rather than crude values provides a more accurate estimation of dietary intake using the BDHQ (50). Therefore, in this study, energy-adjusted values were used to estimate dietary intake. The questionnaire used to estimate barley intake was specifically developed for this study; thus, its validity has not been examined. The validity of this questionnaire should thus be confirmed in the future. To gain a more accurate estimation of dietary intake, combining dietary records with methods, such as 24-h urine collection, serum analysis, and double-labeled water, would be desirable. Second, all participants were affiliated with barley production companies, potentially leading to higher barley consumption by these individuals than by the general Japanese population. However, the median barley intake (3.494 g/1000 kcal) was conducive to daily consumption. Third, the machine learning model employed herein presents challenges in generalizability owing to the anticipated sluggish classification performance when applied to datasets that deviate significantly from this study’s cohort in terms of race and dietary patterns. Future studies should expand the model to cover diverse populations to assess its validity and detect unidentified confounders. Fourth, our understanding of *B. subtilis* intestinal colonization is limited. As a result, the relationship between natto intake and *B. subtilis* presence in feces is unclear. Of note, this study relied on information from rRNA in the 16S region to identify intestinal bacteria, and the role of *B. subtilis* in responders could not be observed. Furthermore, in this study, although 77% of the respondents consumed natto daily, only 34% harbored *Bacillus*. Hence, a further investigation is warranted to elucidate the effects of natto consumption on *B. subtilis* function in the gastrointestinal tract. A longitudinal analysis will be conducted to ascertain the effect of barley on obesity prevention, and potential confounders among respondents lacking *B. subtilis* will be considered. Fifth, as the characteristic bacteria of both responders were butyrate-producing bacteria, the amount of SCFAs must be measured in the fecal samples of participants. However, measuring the metabolites in the fecal samples collected in this study was challenging. To systematically understand how *β*-glucan in barley are metabolized by intestinal bacteria, the SCFAs produced in the gastrointestinal tract must be quantified. Lastly, as this was a cross-sectional study, it cannot be concluded that barley and natto intake directly contribute to the suppression of obesity based on our results. We plan to conduct a long-term, longitudinal study to further investigate the effects of barley and natto consumption on obesity prevention and development.

In summary, our findings highlighted the distinctive characteristics of intestinal bacteria in barley responders to obesity. These individuals were found to exhibit elevated levels of butyrate-producing bacteria and had a higher consumption of natto, potentially enhancing the conversion of dietary fiber into energy within the digestive system. These findings shed new light on the use of barley β-glucan in the gastrointestinal tract and provide novel insights. The concurrent consumption of barley and natto has emerged as a promising nutritional approach for obesity prevention.

## Data Availability

The datasets presented in this study can be found in online repositories. The names of the repository/repositories and accession number(s) can be found in the article/Supplementary material. Other data and analysis codes described in the manuscript of this paper are available from the corresponding author upon appropriate request.
