# Supplementary figures and images for: High barley intake in non-obese individuals is associated with high natto consumption and abundance of butyrate-producing bacteria in the gut: a cross-sectional study
Source: Front Nutr. 2024 Oct 31;11:1434150. doi: 10.3389/fnut.2024.1434150 (PMC11562852; doi:10.3389/fnut.2024.1434150)

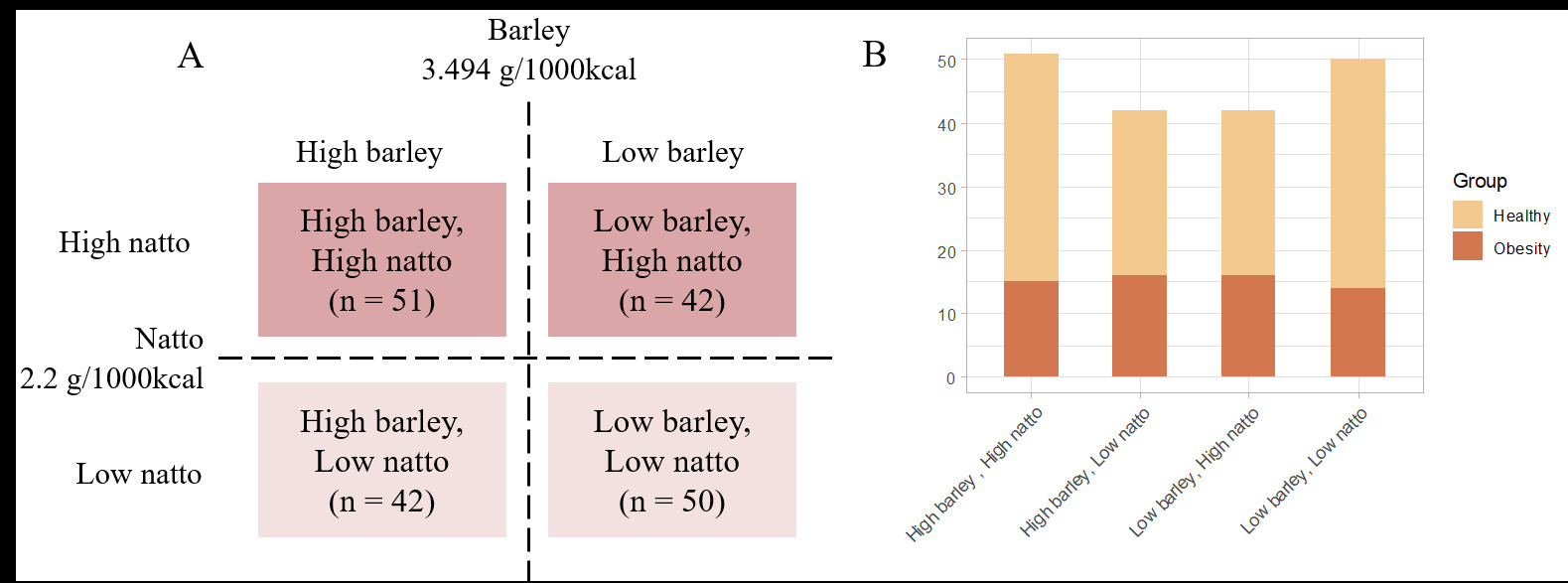

Supplement: Supplementary file 1 [file Image_1.JPEG]
